# Infallible Diagnosis!

**Published:** 1883-02

**Authors:** 


### INFALLIBLE DIAGNOSIS!

A most amusing illustration of the many errors made in diagnosis is furnished by the now famous "senile gangrene" case of ex-Governor Hendricks. That worthy gentleman suffered greatly from a sore on one of his feet. His physician could not cure it, nor (what is of more importance to the scientific regular) could he properly diagnose it. Therefore, a grand consultation was held. Physicians were summoned from the East and the West, from the North and the South, and a grand scientific investigation was forthwith held over that sore foot. The resulting diagnosis was—"senile gangrene;" the cheerful prognosis was—deat[illegible]

In all cases requiring extraordinary ability, Louisville is called upon; and so it was in this case. A learned surgeon from Louisville kindly favored the public with a two-column "interview" on senile gangrene. We can almost see the great tears coursing one another

4

down the cheeks of the tender-hearted reporter as he learned that "science" offered no relief for senile gangrene.

After hearing the decision of the distinguished Dr. "Panglosses" who formed the consultation crew, the ex-Governor folded his hands and awaited the end. Just before that fatal day came, there arrived a country doctor—an old friend of the ex-Governor—to bid him an eternal farewell. With soothing words and tearful handshakings, the *verdant* Æsculapius was about to depart, but before doing so he requested to be allowed to feast his uncultured orbs upon a real, genuine case of scientific "senile gangrene." His request was granted; gently were the coverings removed and the death-dealing "senile gangrene" shown. After gazing at the monster for awhile the rural scientist exclaimed: "Nothing but a d—d boil!!" And so it was. The ex-Governor died not. He is now very lively; but the celebrated surgeons are *not*. Nor does the Louisville Professor interview himself on boils *vs.* senile gangrene.

There is on record only one example of folly so egregious as this, and that was the case of a gentleman who gravely proposed to limit "the dose" *by resolution*. He, too, should consult some rural Æsculapius, and learn of the wonderful cures made by the "non-Hahnnemannic nonsense"—the high potencies.

